# Increased fatty acid availability improves the osteo-anabolic effects of intermittent parathyroid hormone (iPTH) in murine models

**DOI:** 10.1016/j.bonr.2025.101887

**Published:** 2025-11-09

**Authors:** Santosh Thapa, Jake Newberry, S.V.V.S. Ravi Mangu, Ron C.M. Helderman, Ananya Nandy, Cameron Fawcett, Sasidhar Uppuganti, Jeffry S. Nyman, Elizabeth Rendina-Ruedy

**Affiliations:** a Department of Medicine, Division of Clinical Pharmacology, Vanderbilt University Medical Center, Nashville, TN, 37232, USA; b Frank H. Netter M.D. School of Medicine, Quinnipiac University, North Haven, CT, 06473, USA; c Molecular Physiology and Biophysics, Vanderbilt University, Nashville, TN, 37232, USA; d Department of Orthopaedic Surgery, Vanderbilt University Medical Center, 1215 21st Ave. S, Suite 4200, Nashville, TN, 37232, USA; e United States Department of Veterans Affairs, Tennessee Valley Healthcare System, 1310 24th Ave. S., Nashville, TN 37212, USA; f Department of Nutritional Sciences, College of Allied Health, University of Oklahoma Health Campus, 1200 N Stonewall Ave, Oklahoma City, OK, 73117, USA

**Keywords:** Osteoporosis, Bone, Teriparatide, Metabolism, Diet

## Abstract

Osteoporosis is a major public health problem which results in reduced bone mineral density (BMD) and increased fracture risk. Osteoporosis-related fractures often lead to multiple comorbidities which can significantly reduce longevity and diminish one's quality of life. While anabolic agents that increase bone formation, such as parathyroid hormone (PTH), have aided in the management of osteoporosis, patients still experience adverse side-effects along with variations in therapeutic response. Therefore, continued development of refined therapeutic interventions as well as improving efficacy is necessary. Relative to this, the current experiments report on the ability to harness PTH's impact to modulate osteoblast bioenergetic capacity to promote bone formation by supplying fatty acid substrates to meet this energetic demand. To accomplish this, mice were fed a moderately ‘high’ fat diet (2.5×) or control fat, as to not induce metabolic perturbations, while treating with or without PTH for 4 weeks. This dietary regimen resulted in improved bone parameters in mice fed the high fat diet compared to control diet. To directly test the contribution of increased exogenous fatty acid substrates during PTH treatment, we next introduced oleic acid simultaneous to PTH treatment for 4 weeks, and again showed improved skeletal parameters compared to the vehicle-control treated mice. These data support previous publications that demonstrate the osteoanabolic responsiveness of osteoblasts to PTH requires fatty acid substrates. These data further expand on these findings, by providing evidence that PTH efficacy can be improved by supplying exogenous fatty acid substrates, either by dietary or direct interventions.

## Highlights

• A moderately ‘high’ fat diet increased bone formation during concurrent treatment with intermittent parathyroid hormone (iPTH).
• Direct introduction of oleic acid during iPTH treatment increased trabecular bone volume.
• Bone formation by iPTH can be enhanced by coordinating metabolic substrates such as fatty acids during treatment.

## 1 Introduction

Osteoporosis and low bone mass (i.e., osteopenia) are major public health problems, affecting ∼54 million people in the U.S., and nearly half of all adults aged 50 and older (Wright et al., 2017; Aspray and Hill, 2019). Along with the substantial financial burden (∼$19 billion/ year), osteoporosis-related fractures often lead to multiple comorbidities (.e.g., hypertension, deficiency anemias, fluid and electrolyte imbalance), and patients frequently experience diminished quality of life due to immobility, pain, and isolation (Bijelic et al., 2017; Lewiecki et al., 2019; Sattui and Saag, 2014). Until the recent approval of romosuzumab, teriparatide and abaloparatide stood in a class of their own as the only Food and Drug Administration (FDA) approved bone-anabolic agents, both of which exploit parathyroid hormone (PTH) or PTH related protein's (PTHrP) ability to increase bone formation when administered intermittently (Ishtiaq et al., 2015; Liu et al., 2018; Nogues and Martinez-Laguna, 2018; Chew and Clarke, 2017). While these therapeutic options have significantly aided in the management of osteoporosis, some patients still experience undesirable, adverse side-effects (Bauer, 2018; Ferrari et al., 2018). Moreover, they can be cost prohibitory and are frequently prescribed for limited duration. Therefore, continued scientific investigation is needed for the treatment of osteoporosis and/or for improving the efficacy of our current therapies.

As far as the clinical standard of care, intermittent teriparatide (iPTH; 1–34 amino acid fragment) is generally prescribed for severely osteoporotic patients who have sustained one or more fractures (Neer et al., 2001). Due to multiple risk factors including the loss of estrogen during menopause, many patients receiving teriparatide are female (Bauer, 2018; Ferrari et al., 2018). Teriparatide is mostly administered as a daily subcutaneous injection and is commonly prescribed for up to 24 months, albeit recent policy revisions dictate treatment can be extended in patients with high-fracture risk (Hauser et al., 2021; Krege et al., 2022). Following this period, bone formation tends to wane while bone resorption remains elevated, and therefore patients are frequently transitioned to antiresorptive therapies (Chew and Clarke, 2017; Park et al., 2025). One of the main clinical limitations of treatment with teriparatide is the variable responsiveness which can have a wide range (no significant changes to 50 % increase) (Hauser et al., 2021). Interestingly, the reason for limited anabolic response or predictability of patient responsiveness remains unknown. Further underscoring the importance of continued investigation, while teriparatide is administered daily, there is no standard of care regarding time of day and/or dietary considerations.

At the cellular level, PTH-induced bone formation requires an increased workload by the osteoblasts to meet the demands of enhanced secretion of matrix and mineralization vesicles. It is generally appreciated that osteoblasts utilize various energy substrates including glucose, glutamine, and fatty acids, to yield cellular energy or adenosine triphosphate (ATP). Yet because fatty acids yield more energy per molecule when catabolized than glucose, fatty acid oxidation in the mitochondria as a source of adenosine triphosphate (ATP) production is particularly relevant during anabolic states, such as intermittent iPTH. To further support this tenet, it has been demonstrated that in murine models, iPTH-induced cancellous bone formation, requires β-oxidation of fatty acid substrates (Alekos et al., 2023). This paper, along with others (Maridas et al., 2019), elegantly showed that adipocytes can serve as sources of fatty acids, likely supplying the osteoblast with an energy dense substrate during active/ enhanced iPTH-stimulated bone formation. Notably, PTH's pharmacokinetics are relatively short, for example teriparatide peaks at ∼30 min, half-life is ∼1 h, and it's cleared ∼4 h (Satterwhite et al., 2010). Therefore, the window for elevated PTH following intermittent administration *and* substrate availability is relatively narrow and could be a novel variable impacting efficacy. Since little is known about factors enhancing the efficacy of iPTH, we tested whether the osteoanabolic effects of iPTH depended on time of administration, diet, and/or exogenous fatty acids. In doing so, we hypothesize that synchronizing exogenous fatty acid availability, via dietary or direct sources, during iPTH treatment will enhance its osteoanabolic effect, increasing bone mass and bone microstructural quality.

## 2 Materials and methods

### 2.1 Animal details and study designs

For all experiments, C57BL/6N mice were obtained from The Jackson Laboratory (Cat#005304, Jackson Laboratory, Bar Harbor, ME, USA) at 11 weeks of age and allowed to acclimate for one week. Therefore, all studies were initiated on skeletally mature 12-wk old mice. To test whether PTH-responsiveness was impacted by time-of-day of administration female and male mice were housed under a reversed light/ dark cycle (light: 7:00 pm-7:00 am; dark: 7:00 am-7:00 pm) immediately upon arrival and allowed to acclimate for 1 week. Treatments of 100 μg PTH/ kg body weight or vehicle (Veh) control (0.025 % acetic acid in saline) were administered subcutaneously for 4 weeks. PTH dose was selected based on our previous publication specifically demonstrating that PTH-induced osteoblast fatty acid utilization was required for increased bone parameters (Alekos et al., 2023). Mice receiving injections during the dark cycle were given treatments at ∼11:00 am under a red light, while the mice receiving treatment during the light cycle were injected ∼2:00 pm. These timepoints were based on previous data that mice eat most 1–2 h following dark cycle switch, and that serum triglycerides peak ∼3–4 h postprandially (van Diepen et al., 2013; Xie et al., 2014). These mice were fed an AIN-93 M purified diet (Reeves et al., 1993) (Supplemental Table 1).

To determine if diet, namely fat content, affected the osteoanabolic actions of iPTH, both male and female mice 12-wk of age were divided to receive a moderately ‘high’ fat diet (hFD: 25 % kcal from fat; Research Diets INC; D19120501), or a respective control diet (Con: 10 % kcal from fat; Research Diets INC; D12450J; sucrose matched) (Supplemental Table 1) for 4 weeks. These mice were also housed in the reverse light/ dark cycle rooms such that treatments were given during peak food intake (i.e., dark cycle). Again, ∼11:00 am mice were treated with 100 μg PTH/ kg body weight or Veh control (0.025 % acetic acid in saline) subcutaneously.

The final *in vivo* study investigated if a monounsaturated fatty acid affected the anabolism of iPTH. Based on our previous data 12-wk old female mice, under standard light conditions, received a subcutaneous injection of PTH or Veh and intravenous injections oleic acid or vehicle. Thirty minutes after anabolic or control therapy, mice received a tail vein injection of sterile filtered oleic acid-conjugated to bovine serum albumin (BSA) or vehicle of BSA for 4 weeks. Oleic acid (Sigma, O3008; batch# 0000308909) acid was given at a final dose of 5 mg/ kg body weight.

For all experiments human PTH (1–34 amino acid fragment) was obtained from Bachem Americas (Torrance, CA, USA, Cat # NC9752200) and stored in amber glass vials at −80 °C until use. At the termination of the studies, mice were euthanized by exsanguinated via the carotid artery under inhalant isoflurane, and respective tissues were harvested. Mice were weighed weekly throughout the duration of the studies. Regardless of diets, all mice had ad libitum access to food and RO water throughout the study. Mice were group-housed (3–5 per cage) for the experimental period and had access to enrichment huts. Standard housing conditions were maintained within a range of 30–70 % humidity with a set point of 50 %, and a temperature range of 68–76 °F with a set point of 72 °F. All mice were maintained and experiments conducted in accordance with the Guidelines of the Institutional Animal Care and Use Committee (IACUC) at Vanderbilt University Medical Center (VUMC).

### 2.2 Glucose tolerance test

One week prior to the end of the “hFD study” a fasting intraperitoneal glucose tolerance tests (GTT) was performed. Mice were fasted for 6 h before receiving an intraperitoneal (IP) injection of 20 % glucose resuspended in saline for a final dose of 2 g glucose/ kg body weight. Blood glucose was measured before glucose injection (0 min) and 15, 30, 60, and 120 min after injection via tail vein nick using the AlphaTRAK 2 Glucose Meter and AlphaTRAK 2 Test Strips (Zoetis; 71,681–01).

### 2.3 Dual-energy X-ray absorptiometry (DXA)

Dual-energy X-ray absorptiometry (DXA) was performed on all mice at the end of the studies, prior to euthanasia. Briefly, mice were imaged in the prone position using an UltraFocus X-Ray/ DXA (Faxitron, Hologic). Software then provided parameters including bone mineral density (BMD) and body composition such as fat and lean weight. The DXA machine was calibrated using a phantom standard provided by the manufacturer before each scan.

### 2.4 Microcomputed tomography (μCT)

Femurs were cleaned of soft adhering tissues and stored in PBS at −20 °C. Femur length (mm) was measured using digital calipers prior to scans. Bone trabecular and cortical microarchitecture was assessed using an ex-vivo micro-computed tomography (μCT) scanner (μCT 50, Scanco Medical AG, Bruttisellen, Switzerland). The distal femur metaphysis and mid-diaphysis were imaged/ scanned using an X-ray tube peak intensity and current of 70 kVp and 114 mA, respectively, 500 projections per full rotation of the sample, and an integration time of 300 ms, image stacks with an isotropic voxel size of 6 μm were acquired (310 slices each). Analysis of cancellous or trabecular bone was performed defining a region of interest (ROI) 270 μm above the peak of the distal growth plate as to include a region of trabecular bone which extends proximally over 2.7 mm, and segmentation of bone was performed with Gaussian image filter (support of 1.0 and sigma of 0.1) and between a threshold of 350 mgHA/cm^3^ and 3000 mg HA/cm^3^. The attenuation of X-rays was calibrated to a hydroxyapatite (HA) phantom per the guidelines of the manufacturer. The cortical bone was analyzed in a 0.3 mm-long region (50 transverse slices) at the femoral mid-diaphysis with a Gaussian filter (support = 1, and sigma = 0.2), and thresholds of 743.5 and 2229.3 mg HA/cm^3^ were applied to the segment.

### 2.5 Mechanical testing

Intact, hydrated femurs were used for the biomechanical characterization using a three-point bending test protocol. Briefly, the femurs in PBS were thawed to room temperature and mounted on a custom stainless steel test fixture (Catalog no. 2810–410, Instron, Norwood, MA) such that the anterior side rested on the two lower span supports separated by 8 mm and were subjected to tension during the loading phase. A loading fixture, rigidly attached to the Instron actuator (a linear variable differential transducer or LVDT), ramped down at the mid-shaft on the posterior side at a constant rate of 3 mm/min until the sample failed. The lower test fixture that was rigidly attached to a 100 N force transducer (Honeywell Inc., Model 060-N733–04) measured the force on the sample for a given deformation. The force-displacement data were acquired at 50 Hz and subsequently analyzed using a custom processing script in Matlab r2024 (Mathworks Inc., Natick, MA) to compute the structure-related biomechanical properties such as linear elastic stiffness, yield force, ultimate force, and work-to-failure. The force-displacement data in combination with the geometric properties derived from the μCT analysis of the mid-shaft (I_min_, c_min_, Ct.Ar) and using beam theory equations provided the material property estimates of modulus, ultimate bending stress, ultimate moment, and toughness.

### 2.6 Enzyme-linked immunosorbent assays (ELISA)

Following whole blood collection, blood was allowed to clot at room temperature for 30 min and serum was isolated following centrifugation at 4 °C at 3000 x*g* for 10 min. Seum was then frozen at −80 °C until assays were performed. C-terminal telopeptide of type I collagen (CTX-I, RatLaps; AC-06F1) and Procollagen Type I Intact N-terminal Propeptide (PINP; AC-33F1) were determined according to the manufacturers protocol (Immunodiagnostic Systems).

### 2.7 Dynamic and statis bone histomorphometry

To assess dynamic bone changes over a 5-day period, mice were injected with calcein (10 mg/kg body weight) and alizarin (30 mg/kg body weight) at 7 and 2 days prior to euthanasia, respectively. At euthanasia, tibias were harvested, cleaned, and fixed in neutral buffered formalin (NBF) for 48 h. The samples were then dehydrated in a graded series of acetone and infiltrated for 3 days at 4 °C with a solution containing 90 % destabilized methylmethacrylate (MMA), 0.05 % benzoyl peroxide, and 10 % dibutylphthalate. This solution was subsequently replaced with 85 % MMA, 15 % dibutylphthalate, and 4 % benzoyl peroxide for an additional 3–4 days. Following infiltration, the tibias were embedded in MMA and allowed to polymerize. Sections (5 μm thick) were prepared in the transverse plane of the tibia. The region of interest (ROI) was defined as a zone beginning 100 μm distal to the proximal growth plate and extending 800 μm into the metaphyseal region, excluding cortical bone. Dynamic bone formation parameters—mineralizing surface per bone surface (MS/BS), mineral apposition rate (MAR), and bone formation rate per bone surface (BFR/BS)—were quantified by analyzing calcein and alizarin double fluorescent labels. Additional serial sections were stained with tetrachrome and von Kossa to evaluate osteoblasts and osteoclasts within the ROI. Osteoblasts were identified as plump, cuboidal cells lining the trabecular surface (minimum of 3 cells), while osteoclasts were defined as large, multinucleated cells located on trabecular bone surfaces. Bone marrow adipocytes were identified as characteristic empty “ghost cells,” excluding vascular structures and sectioning artifacts. All measurements were analyzed using BioQuant® Osteo (version 18.2.6; BioQuant® Image Analysis Corporation, Nashville, TN). Regions and parameters of analysis adhered to the guidelines established by the Nomenclature Committee of the American Society for Bone and Mineral Research (ASBMR).

### 2.8 Statistical analysis

Data are expressed as the mean ± standard deviation (SD). Statistical analyses were performed using GraphPad Prism 10.4.2. Significant effects of treatment and light cycle, diet, or fat acid as well as their interaction were established employing Two-way ANOVA, followed by post-hoc analysis using Fisher's least significant difference (LSD) tests if warranted. *P* value <0.05 were considered statistically significant, with *p*-values depicted as *p < 0.05, **p < 0.01, ***p < 0.001, ****p < 0.0001.

## 3 Results

**Fig. 1:**
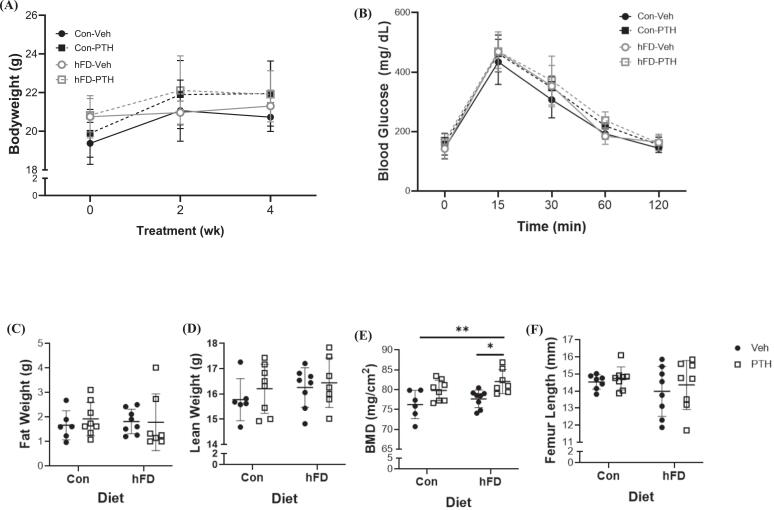
Systemic Metabolism and Bone Characterization Following Dietary Treatment. Twelve-week-old female C57BL/6 N mice were fed a control diet (Con; 10 % kcal from fat) or moderately ‘high’ fat diet (hFD; 25 % kcal from fat) that were treated with vehicle (Veh) or parathyroid hormone (PTH; 5 days/ wk) for a total of 4 weeks. Parameters include (A) bi-weekly bodyweight, and (B) blood glucose following a fasting glucose tolerance test (GTT). Dual-energy X-ray absorptiometry (DXA) results of (C) fat weight (*p-value: diet = 0.9767; treatment = 0.6966; diet x treatment = 0.6195*), (D) lean weight (*p-value: diet = 0.2978; treatment = 0.3637; diet x treatment = 0.7246*), and (E) whole body bone mineral density (BMD) (*p-value: diet = 0.0949; treatment = 0.0.0010; diet x treatment = 0.6974*). (F) Femur length was also determined using digital calipers (*p-value: diet = 0. 2578; treatment = 0.4814; diet x treatment = 0.8375*). All results are expressed as mean ± standard deviation. C–F, each dot represents data from individual animal (*n* = 6–10). Significant differences were established using 2-way analyses of variance (2-way ANOVA) with diet and treatment as independent variables, with multiple comparisons and post-hoc analysis using Fisher's LSD tests. Values of *p* < 0.05 were considered significant, with *p*-values depicted as *p < 0.05, **p < 0.01, ***p < 0.001, ***p < 0.0001.

We first sought to determine whether the timing of iPTH treatment impacted skeletal outcomes as it is recognized mouse models consume food during their dark cycle. Administration of iPTH during the ‘dark’ fed-cycle resulted in increased bone mass represented in higher BMD, whereas no changes in BMD were noted in female mice receiving iPTH during their ‘light’ or sleep cycle (Supplemental Fig. 1A). Serum PINP was significant elevated in mice receiving iPTH regardless of the time of administration (Supplemental Fig. 1B). No changes in serum CTx in female mice (Supplemental Fig. 1C) were detected in either treatment group. This response from iPTH administered during the dark cycle was also demonstrated in male mice with elevated BMD (Supplemental Fig. 1D). Male mice receiving iPTH in both the light and dark cycle demonstrated elevated PINP and CTx (Supplemental Fig. 1E, F). Based on these data, we next tested how modified dietary fat content impacted iPTH, which were carried out under dark cycle administration of PTH to ensure food intake was occurring coincidently with treatments. Importantly, the hFD did not result in obesity or significant weight gain in female or male mice compared to mice receiving the Con diet in either treatment group (Fig. 1A*;* Supplemental Fig. 2A). No overt metabolic disruptions were noted as there were no differences in glucose tolerance, fat or lean weight between diets or treatments in female (Fig. 1B, C, D) or male (Supplemental Fig. 2B, C, D) mice. However, female mice receiving the hFD and PTH treatment demonstrated a higher BMD compared to both Veh groups (Con and hFD) (Fig. 1E). These changes in BMD were not noted in male mice (Supplemental Fig. 2E). There was also no change in femur length due to diet or treatment in either female or male mice (Fig. 1F*;* Supplemental Fig. 2F).

**Fig. 2:**
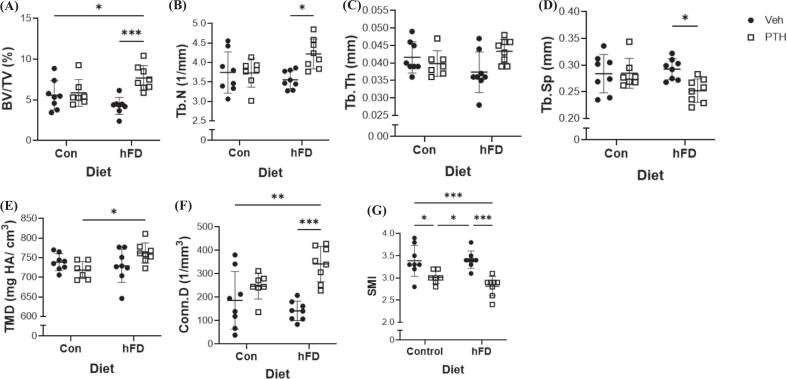
Trabecular Bone Microarchitecture of the Femur Following Diet and PTH Treatments. Micro-computerized tomography (μCT) of femurs isolated from 12-week-old female C57BL/6 N mice fed a control (Con; 10 % kcal from fat) or moderately ‘high’ fat diet (hFD; 25 % kcal from fat) and treated with vehicle (Veh) or parathyroid hormone (PTH; 5 days/ wk) for 4 weeks. Defined regions of interest were within the distal femur metaphysis. Parameters include (A) bone volume over total volume (BV/TV, %), (*p-value: diet = 0.6453; treatment = 0.0023; diet x treatment = 0.0081*); (B) trabecular (Tb.N, mm^−1^), (*p-value: diet = 0.2870; treatment = 0.0295; diet x treatment = 0.0243*); (C) trabecular thickness (Tb.Th, mm), (*p-value: diet = 0.8215; treatment = 0.1989; diet x treatment = 0.0227*); (D) trabecular separation (Tb.Sp, mm), (*p-value: diet = 0.2322; treatment = 0.0520; diet x treatment = 0.0446*); (E) tissue mineral density (TMD, mg hydroxyapatite/ cm^3^), (*p-value: diet = 0.1167; treatment = 0.5621; diet x treatment = 0.0190*);(F) connectivity density (Conn.D; 1/mm^3^), (*p-value: diet = 0.3991; treatment = 0.0001;,diet x treatment = 0.0233*); (G) SMI (*p-value: diet = 0.3181; treatment ≤0.0001; diet x treatment = 0.2029*). Each dot represents data from individual animal (*n* = 7–10). All results are expressed as mean ± standard deviation. Significant differences were established using 2-way analyses of variance (2-way ANOVA) with diet and treatment as independent variables, with multiple comparisons and post-hoc analysis using Fisher's LSD tests. Values of *p* < 0.05 were considered significant, with *p*-values depicted as *p < 0.05, **p < 0.01, ***p < 0.001, ****p < 0.0001.

**Fig. 3:**
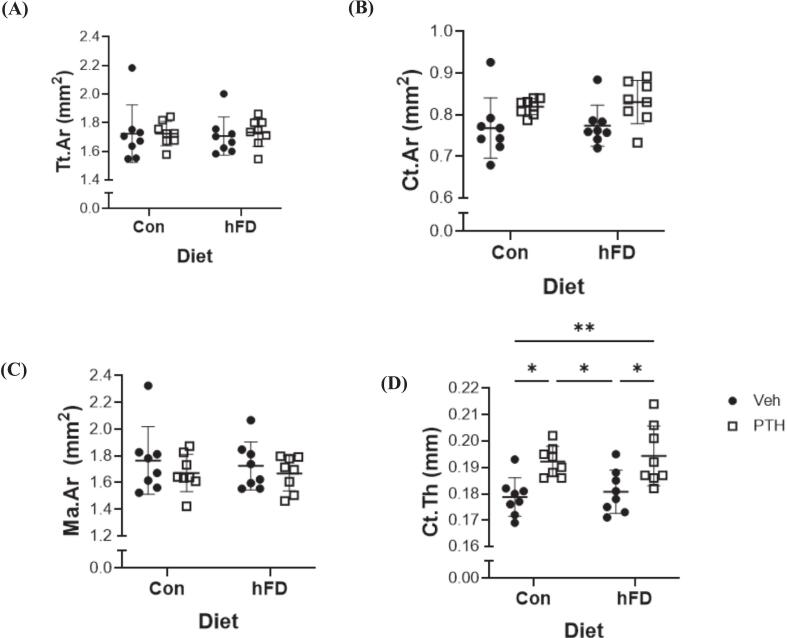
Diet and PTH-related Changes in Cortical Bone Microarchitecture of the Femur. Micro-computerized tomography (μCT) of the mid-diaphysis of femurs isolated from 12-week-old female C57BL/6 N mice fed a control (Con; 10 % kcal from fat) or moderately ‘high’ fat diet (hFD; 25 % kcal from fat) and treated with vehicle (Veh) or parathyroid hormone (PTH; 5 days/ wk) for 4 weeks. Cortical bone analysis of the femur mid-diaphysis includes (A) total cross-sectional area (Tt.Ar, mm^2^), (*p-value: diet = 0.9419; treatment = 0.7814; diet x treatment = 0.7991*); (B) cortical bone area (Ct.Ar; mm^2^) (*p-value: diet = 0.6515; treatment = 0.0066; diet x treatment = 0.8926*); (C) medullary or marrow area (Ma.Ar, mm^2^), (*p-value: diet = 0.7334; treatment = 0.2590; diet x treatment = 0.7771*); (D) cortical thickness (Ct.Th, mm), (*p-value: diet = 0.4927; treatment ≤0.0001; diet x treatment = 0.9833*). Each dot represents data from individual animal (n = 7–10). All results are expressed as mean ± standard deviation. Significant differences were established using 2-way analyses of variance (2-way ANOVA) with diet and treatment as independent variables, with multiple comparisons and post-hoc analysis using Fisher's LSD tests. Values of *p* < 0.05 were considered significant, with *p*-values depicted as *p < 0.05, **p < 0.01, ***p < 0.001, ****p < 0.0001.

Following 4 weeks of PTH treatment, which is considered the early side of detecting skeletal changes in murine models, we did not detect differences in trabecular bone volume fraction between female mice treated with Veh or PTH fed the Con diet (Fig. 2A). Interestingly, female mice that received the hFD with PTH treatment demonstrated an increase in bone volume fracture compared to mice on the hFD receiving Veh treatment as well as Veh-treated mice on the Con diet (Fig. 2A). These changes were further reflected as an increase in trabecular number in mice fed the hFD compared to mice receiving the Veh treatment, while no changes were detected in mice fed the Con diet (Fig. 2B). There were no changes in trabecular thickness amongst any of the groups (Fig. 2C), while trabecular separation was reduced in mice on the hFD treated with PTH compared to hFD-fed mice receiving the Veh (Fig. 2D). No changes were detected in trabecular tissue mineral density between treatments within a given diet (Fig. 2E). Connectivity density was significantly increased only in the female mice receiving the hFD and PTH treatment compared to Veh treated, both on the Con and hFD (Fig. 2F). Structure model index indicated that PTH treatment in both diets, Con and hFD, resulted in more parallel plates of trabeculae (Fig. 2G). No changes were detected in cortical total cross-sectional area (Tt.Ar), cortical area (Ct.Ar), or marrow area (Ma.Ar) between diet or treatment groups (Fig. 3A, B, C). Cortical thickness, however, increased in response to PTH treatment compared to Veh in both the Con and hFD groups (Fig. 3D). In male mice, no changes were demonstrated within diets between treatments in trabecular bone volume fraction (Supplemental Fig. 3A). Surprisingly, PTH treatment in both the Con and hFD groups reduced trabecular number, with no changes in trabecular thickness, and increased trabecular separation (Supplemental Fig. 3B, C, D). No changes were detected in trabecular connectivity density in male mice within treatments or diets. PTH treatment in male mice also resulted in in more rod-like trabecular struts (Supplemental Fig. 3F) within both dietary regimens. No changes were detected in cortical parameters following PTH treatment compared to Veh or between dietary groups (Supplemental Fig. 3H, I, J, K).

Dynamic bone histomorphometry was consistent with these microarchitectural bone data as mineralizing surface and bone formation rates were the greatest in female mice receiving a hFD and PTH treatment (Table 1). The number of osteoblasts increased in PTH-treated groups while osteoclasts were only increased in mice receiving the PTH treatment during control diet feeding (Table 1). Bone marrow adiposity was not changing in any treatment groups with either diet (Table 1), consistent with the ‘normal’ systemic metabolic phenotype (i.e., no change in body weight, fat weight, and/or glucose tolerance in Fig. 1).

**Table 1:** Dynamic and static bone histomorphometry of the proximal tibia following dietary treatment with or without PTH.

	Con-Veh	Con-PTH	hFD-Veh	hFD-PTH	*diet*	*treatment*	*diet x treatment*
MS/BS (%)	13.29 ± 1.86^C^	17.1 ± 1.84^B^	13.99 ± 1.89^C^	25.29 ± 1.98^A^	*<0.0001*	*<0.0001*	*<0.0001*
MAR (μm/day)	1.14 ± 0.16^B^	1.87 ± 0.28^A^	1.22 ± 0.22^B^	2.21 ± 0.35^A^	*0.0363*	*<0.0001*	*0.1840*
BFR/BS (μm^3^/μm^2^/day)	0.27 ± 0.03^B^	0.36 ± 0.11^B^	0.30 ± 0.07^B^	0.75 ± 0.08^A^	*<0.0001*	*<0.0001*	*<0.0001*
N.Ob/BS (N/mm)	26.66± 3.04^B^	35.66 ± 4.98^A^	27.16 ± 2.12^B^	33.24 ± 4.37^A^	*0.4821*	*<0.0001*	*0.2851*
N.Oc/BS (N/mm)	2.58 ± 0.44^B^	8.4 ± 0.53^A^	2.80 ± 0.79^B^	3.04 ± 0.76^AB^	*0.2148*	*0.0028*	*0.0326*
BMAd.V/TV (%)	1.00 ± 0.35	0.80 ± 0.12	0.91 ± 0.21	0.96 ± 0.14	*0.7063*	*0.3493*	*0.1203*

Twelve-week-old female C57BL/6 N mice were fed a control diet (Con; 10 % kcal from fat) or moderately ‘high’ fat diet (hFD; 25 % kcal from fat) that were treated with vehicle (Veh) or parathyroid hormone (PTH; 5 days/ wk) for a total of 4 weeks. Bone histomorphometry was performed on the proximal tibia metaphysis. Dynamic parameters include mineralizing surface per bone surface (MS/BS), mineral apposition rate (MAR), bone formation rate per bone surface (BFR/ BS); while static parameters include number of osteoblasts per BS (N.Ob./BS), number of osteoclasts per BS (N.Oc/BS), and bone marrow adipocyte volume per total volume (BMAd.V/TV). All results are expressed as mean ± standard deviation (*n* = 7–8). Significant differences were established using 2-way analyses of variance (2-way ANOVA) with diets and treatment as independent variables, with multiple comparisons and post-hoc analysis using Fisher's LSD tests (*p-values are italicized*). Rows that do not share the same superscript are significantly different.

**Fig. 4:**
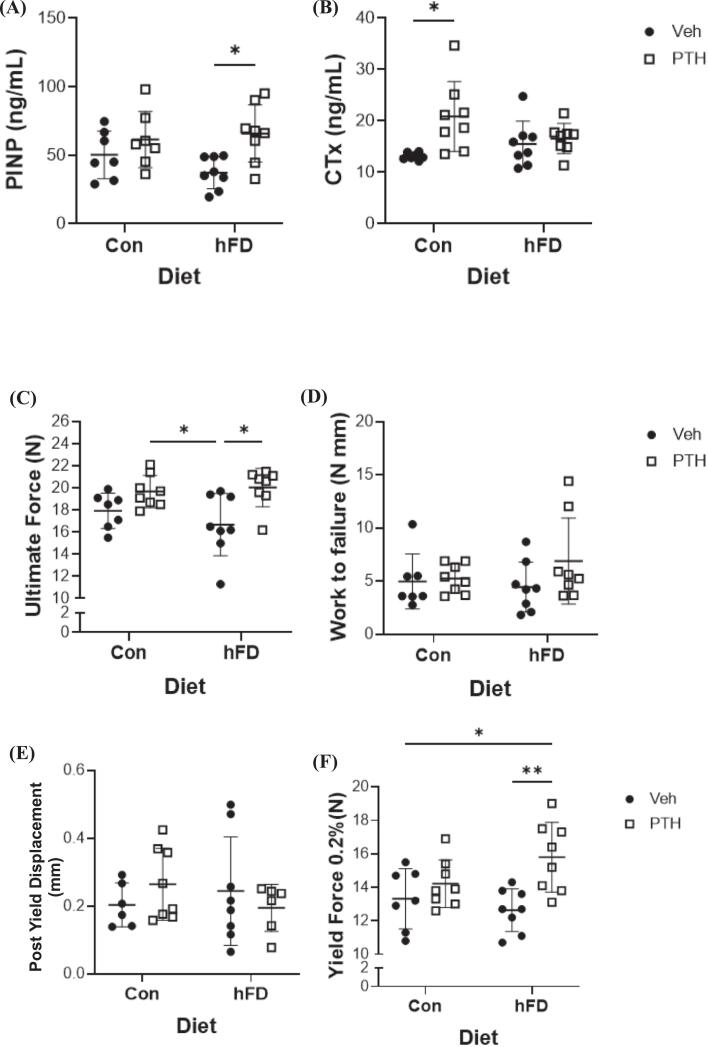
Bone Turnover Markers and Bone Biomechanics Following PTH and/or Dietary Treatments. Female mice were fed a control (Con; 10 % kcal from fat) or moderately ‘high’ fat diet (hFD; 25 % kcal from fat) and treated with vehicle (Veh) or parathyroid hormone (PTH; 5 days/ wk) for 4 weeks. Serum was isolated and used for bone turnover markers including (A) procollagen I intact N-terminal or PINP, (*p-value: diet = 0.5106; treatment = 0.0053; diet x treatment = 0.1942*) and (B) C-terminal telopeptide or CTx, (*p-value: diet = 0.5566; treatment = 0.0098; diet x treatment = 0.0456*). Each dot represents data from individual animal (n = 7–8). All results are expressed as mean ± standard deviation. Three-point bending was also performed on the femur, these parameters include (C) ultimate force (N) (*p-value: diet = 0.5375; treatment = 0.0013; diet x treatment = 0.2673*); (D) work to failure (KJ/m^2^) (*p-value: diet = 0.5743; treatment = 0.1818; diet x treatment = 0.2835*); (E) post yield displacement (mm), (*p-value: diet = 0.9380; treatment = 0.8485; diet x treatment = 0.6015*); and (F) yield force (N) (*p-value: diet = 0.4550; treatment = 0.0022; diet x treatment = 0.0703*). Each dot represents data from individual animal (*n* = 5–8). All results are expressed as mean ± standard deviation. Significant differences were established using 2-way analyses of variance (2-way ANOVA) with diet and treatment as independent variables, with multiple comparisons and post-hoc analysis using Fisher's LSD tests. Values of *p* < 0.05 were considered significant, with *p*-values depicted as *p < 0.05, **p < 0.01, ***p < 0.001, ****p < 0.0001.

Bone turnover markers demonstrate no significant changes in PINP between treatments in female mice fed a Con diet, however, mice fed a hFD demonstrated elevated PINP following PTH treatment compared to Veh (Fig. 4A). Interestingly, CTx a marker of bone resorption, which is frequently coupled with PTH-induced bone formation, was only elevated in PTH-treated mice on the Con diet compared to Veh, while no changes were observed in hFD fed mice amongst treatment (Fig. 4B). Serum PINP was increased in male mice only in the group treated with PTH receiving the hFD compared to Veh, while CTx was increased in response to PTH compared to Veh in both Con and hFD fed mice (Supplemental Fig. 4A, B). Hence, the modest changes in bone microarchitecture in the male mice could be attributed to increased bone turnover, with bone formation keeping pace with resorption. Based on these data, the remaining datasets and experiments included only female mice.

Relative to how these changes translated to biomechanical properties of the femur, we demonstrate an increase in ultimate force in female mice fed the hFD treated with PTH relative to the Veh (Fig. 4C). Ultimate force was higher in the PTH treated mice on the Con diet, but only relative to the Veh-hFD mice (Fig. 4C). There were no significant changes in work to failure and post yield displacement between either treatment or diet (Fig. 4D, E). Yield force was also increased in the PTH treated mice receiving the hFD compared to Veh hFD and Con mice (Fig. 4F).

**Fig. 5:**
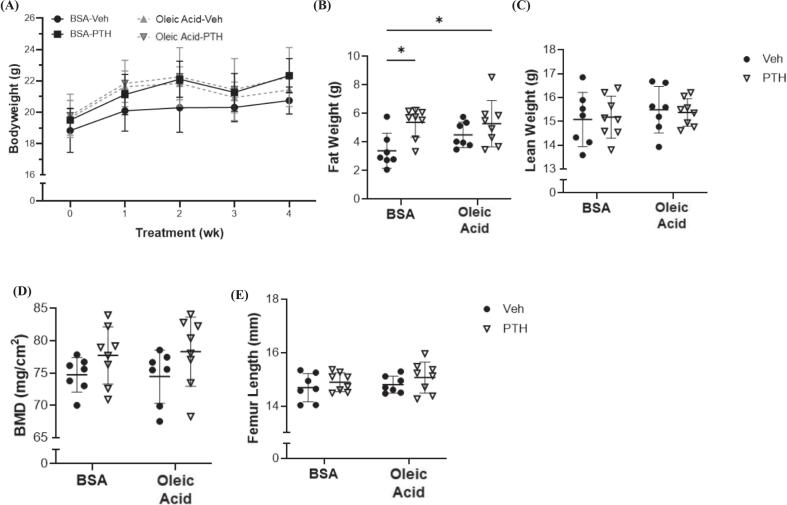
Oleic Acid and PTH Treatment Effects on Systemic Metabolism and Bone. Twelve-week-old female C57BL/6 N mice were treated (injected) with bovine serum albumin (BSA) or oleic acid-BSA and further treated with vehicle (Veh) or parathyroid hormone (PTH; 5 days/ wk) for a total of 4 weeks. Parameters include (A) weekly bodyweights; dual-energy X-ray absorptiometry (DXA) results of (B) fat weight (*p-value: injection = 0.2715; treatment = 0.005; injection x treatment = 0.1946*), (C) lean weight (*p-value: injection = 0.3663; treatment = 0.9827; injection x treatment = 0.7529*), and (D) whole body bone mineral density (BMD) (*p-value: injection = 0.9302; treatment = 0.0387; injection x treatment = 0.7891*). (F) Femur length was also determined by using digital calipers (*p-value: injection = 0.4048; treatment = 0.1724; injection x treatment = 0.8695*). All results are expressed as mean ± standard deviation. C–F, each dot represents data from individual animal (*n* = 6–8). Significant differences were established using 2-way analyses of variance (2-way ANOVA) with diet and treatment as independent variables, with multiple comparisons and post-hoc analysis using Fisher's LSD tests. Values of *p* < 0.05 were considered significant, with *p*-values depicted as *p < 0.05, **p < 0.01, ***p < 0.001, ***p < 0.0001.

Given these hFD results, we next sought to test whether directly introducing a long chain fatty acid, oleic acid, simultaneous to PTH treatment would impact skeletal outcomes in female mice. As expected, there were no differences in bodyweight between groups receiving the oleic acid or BSA control treated with Veh or PTH (Fig. 5A). Not expected, was the significant increase in fat weight following PTH treatment in mice administered both the BSA control and oleic acid (Fig. 5B). There were no changes in lean body weight between any of the groups (Fig. 5C). There were also no changes in whole body BMD or femur length amongst any of the groups (Fig. 5D, E).

**Fig. 6:**
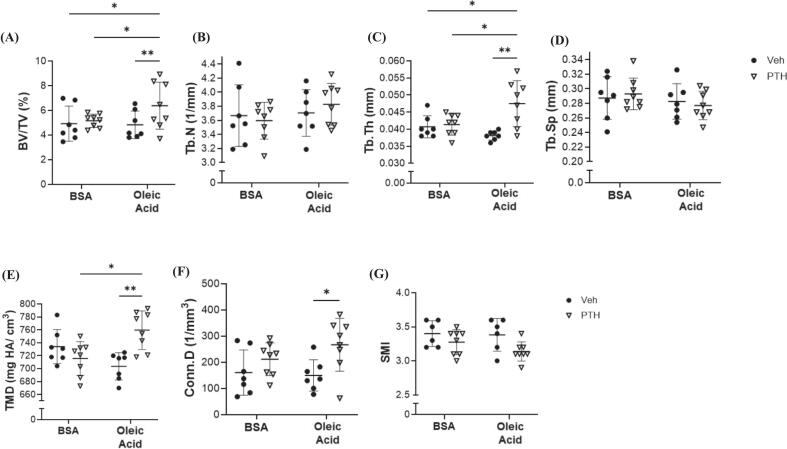
Trabecular Bone Microarchitecture of the Femur Following Treatment of Oleic Acid and/or PTH. Micro-computerized tomography (μCT) of femurs isolated from 12-week-old female C57BL/6 N mice treated, intravenously, with bovine serum albumin (BSA) or oleic acid-BSA and further treated with vehicle (Veh) or parathyroid hormone (PTH; 5 days/ wk) for a total of 4 weeks. Parameters include (A) bone volume over total volume (BV/TV, %), (*p-value: injection = 0.0583; treatment = 0.0114; injection x treatment = 0.0373*); (B) trabecular (Tb.N, mm^−1^), (*p-value: injection = 0.2770; treatment = 0.8366; injection x treatment = 0.4359*); (C) trabecular thickness (Tb.Th, mm), (*p-value: injection = 0.2569; treatment = 0.0030; injection x treatment = 0.0087*); (D) trabecular separation (Tb.Sp, mm), (*p-value: injection = 0.2470; treatment = 0.9878; injection x treatment = 0.5265*); (E) tissue mineral density (TMD, mg hydroxyapatite/ cm^3^), (*p-value: injection = 0.4895; treatment = 0.0611; injection x treatment = 0.0006*); (F) connectivity density (Conn.D; 1/mm^3^), (*p-value: injection = 0.4565; treatment = 0.0079; injection x treatment = 0.2674*); (G) SMI (*p-value: injection = 0.2919; treatment = 0.0160; injection x treatment = 0.4067*). Each dot represents data from individual animal (*n* = 5–8). All results are expressed as mean ± standard deviation. Significant differences were established using 2-way analyses of variance (2-way ANOVA) with diet and treatment as independent variables, with multiple comparisons and post-hoc analysis using Fisher's LSD tests. Values of *p* < 0.05 were considered significant, with *p*-values depicted as *p < 0.05, **p < 0.01, ***p < 0.001, ****p < 0.0001.

**Fig. 7:**
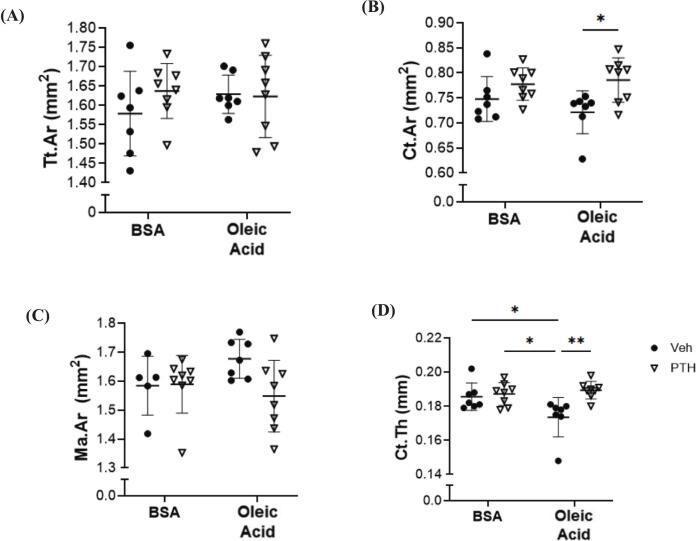
Cortical Bone Microarchitecture of the Femur Following Treatment of Oleic Acid and/or PTH. Micro-computerized tomography (μCT) of femurs isolated from 12-week-old female C57BL/6 N mice treated with bovine serum albumin (BSA) or oleic acid-BSA and further treated with vehicle (Veh) or parathyroid hormone (PTH; 5 days/ wk) for a total of 4 weeks. Cortical bone analysis of the femur mid-diaphysis includes (H) total cross-sectional area (Tt.Ar, mm^2^), (*p-value: injection = 0.5781; treatment = 0.4193; injection x treatment = 0.3250*); (I) cortical bone area (Ct.Ar; mm^2^) (*p-value: injection = 0.5438; treatment = 0.0045; injection x treatment = 0.2625*); (J) medullary or marrow area (Ma.Ar, mm^2^), (*p-value: injection = 0.5110; treatment = 0.1232; injection x treatment = 0.0987*); (K) cortical thickness (Ct.Th, mm), (*p-value: injection = 0.1097; treatment = 0.0067; injection x treatment = 0.0228*). Each dot represents data from individual animal (n = 5–8). All results are expressed as mean ± standard deviation. Significant differences were established using 2-way analyses of variance (2-way ANOVA) with diet and treatment as independent variables, with multiple comparisons and post-hoc analysis using Fisher's LSD tests. Values of p < 0.05 were considered significant, with *p*-values depicted as *p < 0.05, **p < 0.01, ***p < 0.001, ****p < 0.0001.

Microarchitecture of the femur revealed that female mice treated with both PTH and oleic acid had an increased trabecular bone volume fraction compared to all other groups (Fig. 6A). There were no changes in trabecular number, however, trabecular struts were thicker following PTH and oleic acid treatment (Fig. 6B and C). No changes were detected in trabecular separation between treatments or with/ without oleic acid administration (Fig. 6D*)* Trabecular TMD and Conn.D were also increased in mice treated with oleic acid and PTH compared to Veh, while PTH did not affect those parameters in BSA treated mice (Fig. 6E, F, G). No changes were demonstrated in cortical cross-sectional area or marrow area between any groups, but cortical area and thickness were increased in response to PTH only in mice receiving the oleic acid, when compared to Veh treatment (Fig. 7H, I, J, K). Dynamic parameters of bone formation including mineralizing surface and bone formation rates were the highest in mice receiving the oleic acid during PTH treatment (Table 2). No changes were detected in osteoblast numbers amongst either injection or treatments, but osteoclast numbers were higher in the PTH group receiving the oleic acid injection (Table 2). There were no changes in bone marrow adiposity (Table 2).

**Table 2:** Dynamic and static bone histomorphometry following injections of BSA or OA during PTH treatment.

	BSA-Veh	BSA-PTH	Oleic Acid-Veh	Oleic Acid-PTH	*injection*	*treatment*	*injection x treatment*
MS/BS (%)	15.51 ± 2.36^B^	15.45 ± 3.03^B^	16.35 ± 2.13^AB^	19.13 ± 2.00^A^	*0.0131*	*0.1209*	*0.1084*
MAR (μm/day)	0.34 ± 0.17	1.01 ± 0.27	0.91 ± 0.22	1.23 ± 0.32	*0.2553*	*0.0371*	*0.1682*
BFR/BS (μm^3^/μm^2^/day)	0.22 ± 0.05^B^	0.23 ± 0.04^B^	0.21 ± 0.05^B^	0.31 ± 0.07^A^	*0.0808*	*0.0083*	*0.0205*
N.Ob/BS (N/mm)	28.03 ± 2.10	28.48 ± 3.52	27.60 ± 1.77	28.95 ± 2.26	*0.9797*	*0.3009*	*0.6031*
N.Oc/BS (N/mm)	2.33 ± 0.43^B^	3.28 ± 0.89^B^	2.40 ± 0.48^B^	4.72 ± 1.20^A^	*0.0134*	*<0.0001*	*0.0237*
BMAd.V/TV (%)	1.00 ± 0.19	0.79 ± 0.16	0.91 ± 0.15	0.89 ± 0.18	*0.9506*	*0.0645*	*0.1623*

Twelve-week-old female C57BL/6 N mice were treated (injected) with bovine serum albumin (BSA) or oleic acid as ‘injections’ and further treated with vehicle (Veh) or parathyroid hormone (PTH; 5 days/ wk) for a total of 4 weeks. Bone histomorphometry was performed on the proximal tibia metaphysis. Dynamic parameters include mineralizing surface per bone surface (MS/BS), mineral apposition rate (MAR), bone formation rate per bone surface (BFR/ BS); while static parameters include number of osteoblasts per BS (N.Ob./BS), number of osteoclasts per BS (N.Oc/BS), and bone marrow adipocyte volume per total volume (BMAd.V/TV). All results are expressed as mean ± standard deviation (*n* = 7–8). Significant differences were established using 2-way analyses of variance (2-way ANOVA) with injections and treatment as independent variables, with multiple comparisons and post-hoc analysis using Fisher's LSD tests (*p-values are italicized*). Rows that do not share the same superscript are significantly different.

**Fig. 8:**
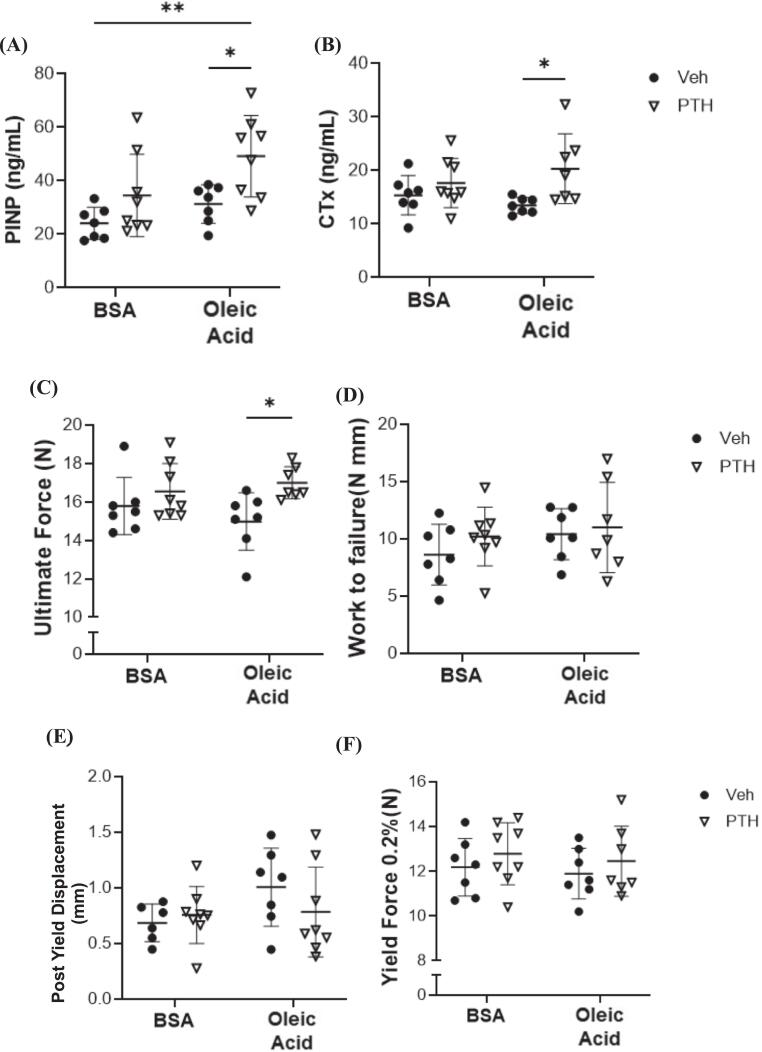
Bone Turnover Markers and Bone Biomechanics Following Oleic Acid (OA) Treatment and/or PTH. Twelve-week-old female C57BL/6 N mice were treated (injected) with bovine serum albumin (BSA) or oleic acid-BSA and further treated with vehicle (Veh) or parathyroid hormone (PTH; 5 days/ wk) for a total of 4 weeks. Serum was isolated and used for bone turnover markers including (A) procollagen I intact N-terminal or PINP, (*p-value: injection = 0.0203; treatment = 0.0037; injection x treatment = 0.4056*) and (B) C-terminal telopeptide or CTx, (*p-value: injection = 0.8300; treatment = 0.0105; injection x treatment = 0.1799*). Each dot represents data from individual animal (*n* = 7–8). All results are expressed as mean ± standard deviation. Three-point bending was also performed on the femur, these parameters include (C) ultimate force (N) (*p-value: injection = 0.7308; treatment = 0.0106; injection x treatment = 0.2255*); (D) work to failure (KJ/m^2^) (*p-value: injection = 0.2446; treatment = 0.3280; injection x treatment = 0.6496*); (E) post yield displacement (mm), (*p-value: injection = 0.1529; treatment = 0.5262; injection x treatment = 0.2248*); and (F) yield force (N) (*p-value: injection = 0.5466; treatment = 0.2612; injection x treatment = 0.9650*). Each dot represents data from individual animal (n = 5–8). All results are expressed as mean ± standard deviation. Significant differences were established using 2-way analyses of variance (2-way ANOVA) with diet and treatment as independent variables, with multiple comparisons and post-hoc analysis using Fisher's LSD tests. Values of p < 0.05 were considered significant, with *p*-values depicted as *p < 0.05, **p < 0.01, ***p < 0.001, ****p < 0.0001.

Consistent with the bone microarchitectural changes, serum PINP and CTx were elevated in mice treated with both PTH and oleic acid compared to both Veh groups, no changes were noted within BSA treatments (Fig. 8A, B). Biomechanically, PTH treatment with oleic acid resulted in an increase in ultimate force compared to Veh treatment with oleic acid, while no changes were detected in BSA treated groups (Fig. 8C). No changes were demonstrated in work to failure, post yield displacement, or yield force amongst any treatment group (Fig. 8D, E, F*)*.

## 4 Discussion

This study demonstrates that the osteoanabolic actions of intermittent PTH treatment can be enhanced with exogenous fatty acid availability. These data expand on prior publications that establish osteoblasts utilize long chain fatty acid β-oxidation as an energy substrate to support the increase in bone formation (Alekos et al., 2023; Maridas et al., 2019). While these previous studies focused on adipocytes, peripheral and/or bone marrow adipocytes, as a source for these fatty acids, the current studies compliment these data by providing support that exogenous sources of fatty acids can also promote PTH-induced bone formation

Within the context of the current studies we first recognized the potential limitation of ensuring adequate availability of dietary-derived fatty acids given the nocturnal nature of laboratory mice (Ellacott et al., 2010; Peirson et al., 2018). With this concept in mind, along with the short half-life of PTH, we surmised that fatty acid availability could be limited during the light/ sleep cycle that mice are typically treated during, due to convenience. Our initial pilot study indeed demonstrated that the timing of PTH administration was an important factor to consider when performing preclinical PTH studies. Administering PTH during the dark-cycle, or active feeding cycle of mice, resulted in a significant increase in bone parameters compared to mice receiving PTH during their light-cycle. Circulating, endogenous PTH has also been demonstrated to be lower during the ‘awake’ phase, presumably as mice are actively eating and serum calcium levels are sustained (Jubiz et al., 1972). These data are somewhat contradictory to a clinical study which demonstrated similar response to teriparatide treatment whether it was received in the morning or evening in BMD of the radius and femur (Michalska et al., 2012). Moreover, they also report that teriparatide treatment received in the morning resulted in a significantly higher BMD of the lumbar spine compared to evening treatment (Michalska et al., 2012), which could suggest exogenous dietary factors are not as important in humans. Interestingly, within the same study, serum markers were more consistent with our results, as serum PINP was elevated after 6 months of evening treatment with teriparatide compared to morning treatment (Michalska et al., 2012). Notable, fat oxidation is highest in the early mornings in humans (Rubio-Valles et al., 2025), and teriparatide treatment could theoretically synchronize with enhanced osteoblast fatty acid oxidation as well, primarily from endogenous sources, thus resulting in increased bone formation. A limitation in our study is that the mice receiving PTH during their active-wake cycle (dark) were also likely loading their skeleton and other metabolic substrates were available (i.e., glucose and glutamine). While we are unable to disentangle this data, we further tested whether we could administer PTH during the dark cycle while ingesting a moderately ‘high’ fat diet (2.5× that of control). As with many dietary studies, to increase exogenous fat, carbohydrates were reduced. Importantly, mice recieving this hFD did not demonstrate an increase in body weight, fasting blood glucose, or glucose intolerance suggesting our diet did not disrupt systemic metabolism. It's expected that these dietary fatty acids were esterified as triacylglycerols (TAGs) and packaged into chylomicrons within the gastrointestinal tract (GI), prior to circulating throughout the body, but would directly reach the bone as has been demonstarted previously (Niemeier et al., 2008). At this stage osteoblasts would need a mechanism to hydrolyze chylomicron bound TAGs, to release fatty acids for cellular uptake. Interestingly, while it has not been extensively studied, osteoblasts express high levels of hepatic lipase (*Lipc*) (Bartelt et al., 2014), which if fact acts as an extracellular lipase to do exactly this. Therefore, our data supports that circulating exogenous dietary sources of moderate amounts of fatty acids, packaged within chylomicrons, can enhance PTH osteoanabolic actions. While we aimed to synchronize PTH treatment when plasma free fatty acids were highest following hFD ingestion in our murine model, i.e., following food ingestion during their active/ dark cycle, we did not monitor every mouse to confirm this. Finally, while we did note differences in response to PTH and/or dietary modifications between males and females, it was beyond the scope of the current project to tease this apart. However, given the extensive data describing differences in metabolic rates between sexes, in both mice and humans (de Souza et al., 2022; Arciero et al., 1993), this would be an interesting area of exploration within the context of osteoporosis treatments.

As a complement to this hFD study we directly introduced oleic acid bound to BSA along with PTH treatment to circumvent limitations and confounding variables related to our dietary treatment. Unexpectedly, BSA treatment along with PTH resulted in higher fat weight compared to control group. This was not overtly pronounced or reflected in whole body weight. Even with this modest difference in fat weight, no skeletal parameters were different within the BSA group. While we expected to detect increases in serum markers of bone turnover, we were pleasantly surprised to detect significant improvement in skeletal parameters in mice treated with oleic acid and PTH compared to oleic acid-Veh. These data support that direct introduction of oleic acid along with simultaneous PTH treatment, even with its short half-life, can support osteoblastic bone formation, consistent with the hFD study and previous reports (Alekos et al., 2023). While we selected oleic acid based on its abundance in the ‘hFD’ which includes additional fat from lard (Rendina-Ruedy and Smith, 2016; Rendina-Ruedy and Smith, 2022) and its positive impact on mitochondrial function (Nakamura et al., 2014; Gillet et al., 2015; Yuzefovych et al., 2010), this study opens the possibility to test various fatty acid substrates impact on promoting bone formation during PTH treatment. Regarding our hFD, fats that were increased primarily included long chain fatty acids including oleic acid, along with palmitic acid, stearic acid, myristic acid, linoleic and α-linolenic acid. As such, these would be interesting to test further/ directly. Finally, it's possible that different length fatty acids and/or saturation status can be explored for supplementation with PTH treatment. For example, omega-3 polyunsaturated fatty acids (PUFA), commonly found in fish oil, has been shown clinically to be associated with positive outcomes for bone health (Mangano et al., 2013). Furthermore, a recent animal study has described omega-3 PUFA's ability to protect against obesity-induced reduction in skeletal health (Benova et al., 2023). Therefore, enhancing the availability of long-chain fatty acids, in this case oleic acid, increased PTH-related bone turnover, ultimately increasing bone volume, and trending towards increased strength. As a final note, we acknowledge that our data regarding treatment with PTH for 4 weeks and not detecting an osteoanabolic response is different than some previous publication (Jilka et al., 1999; Bellido et al., 2005; Delgado-Calle et al., 2017). While we cannot fully address these differences, we have noted three primary differences including dosing regimen (5 days/ week vs. 7 days/ week), age at treatment (3 vs. 4 vs. 5 mo old), and potential differences in mouse strains and/or sub-strains used (C57BL/6N vs. C57BL/6Nhsd, or not reported/ defined) (Jilka et al., 1999; Bellido et al., 2005). Therefore, given that our experiments use the same dose of PTH, it is difficult to directly compare these results.

Collectively, our data demonstrates that the modulation of fatty acid substrates during PTH treatment improves skeletal outcomes. These data further provide foundation for further studies to expand on the ability of dietary modifications to improve in the outcomes of osteoporosis therapies, beyond that of calcium and vitamin D.

The following are the supplementary data related to this article.

Fig. S1 Determination of Time-Related Efficacy during PTH treatment. Twelve-week-old C57BL/6N fe/male mice were fed on an AIN-93M purified diet and subcutaneously treated with vehicle (Veh; closed dark circle) or parathyroid hormone (PTH; closed dark square); 5 days/ wk for a total of 4 weeks. Mice received injections during their light cycle (Light) or dark cycle (Dark) under a red light. Dual-energy X-ray absorptiometry (DXA) results of bone mineral density (A) and (D) for female and male mice, respectively. Serum was isolated and used for bone turnover markers including procollagen I intact N-terminal or PINP (B and E) and C-terminal telopeptide or CTx (C and F) for female and male mice, respectively. Each dot represents data from individual animal where *n* = 7–11. All results are expressed as mean ± standard deviation. Significant differences were established using 2-way analyses of variance (2-way ANOVA) with diet and treatment as independent variables, with multiple comparisons and post-hoc analysis using Fisher's LSD tests. Values of *p* < 0.05 were considered significant, with *p*-values depicted as *p < 0.05, **p < 0.01, ***p < 0.001, ****p < 0.0001. Fig. S1

Fig. S2 Systemic Metabolism and Bone Characterization Following Dietary Treatment in Male Mice. Twelve-week-old male C57BL/6N mice were fed on a control diet (Con; 10 % kcal from fat) or moderately ‘high’ fat diet (hFD; 25 % kcal from fat) and treated with vehicle (Veh) or parathyroid hormone (PTH; 5 days/ wk) for a total of 4 weeks. Parameters include (A) bi-weekly bodyweight and (B) fasting, oral glucose tolerance test (GTT) a week prior to the study termination. Dual-energy X-ray absorptiometry (DXA) results of (C) fat weight, (D) lean weight, and (E) whole body bone mineral density (BMD). (F) Femur length was determined using digital calipers. All results are expressed as mean ± standard deviation. C–F, each dot represents data from individual animal (*n* = 6–10) treated with Veh (closed circle) and PTH (open square). Significant differences were established using 2-way analyses of variance (2-way ANOVA) with diet and treatment as independent variables, with multiple comparisons and post-hoc analysis using Fisher's LSD tests. Values of *p* < 0.05 were considered significant, with *p*-values depicted as *p < 0.05, **p < 0.01, ***p < 0.001, ****p < 0.0001. Fig. S2

Fig. S3 Bone Microarchitecture in Male Mice Following PTH treatment during Dietary Modifications. Micro-computed tomography (μCT) analysis from the femur metaphysis of trabecular and cortical bone analysis of the femur mid-diaphysis in male C57BL/6 N mice fed a control (Con; 10 % kcal from fat) or moderately ‘high’ fat diet (hFD; 25 % kcal from fat) and treated with vehicle (Veh; closed circle) or parathyroid hormone (PTH; open square) for 5 days/wk. throughout 4 weeks. Trabecular parameters (A–G) include trabecular bone volume fraction, trabecular number (Tb.N), trabecular thickness (Tb.Th), trabecular separation (Tb.Sp), tissue mineral density (TMD), connectivity density (Conn.D), and structural model index (SMI). Cortical bone properties (H–K) include total cross-sectional area (Tt.Ar), cortical area (Ct.Ar), marrow area (Ma.Ar), and cortical thickness (Ct.Th). Each dot represents data from individual animal where *n* = 7–10. All results are expressed as mean ± standard deviation. Significant differences were established using 2-way analyses of variance (2-way ANOVA) with diet and treatment as independent variables, with multiple comparisons and post-hoc analysis using Fisher's LSD tests. Values of *p* < 0.05 were considered significant, with *p*-values depicted as *p < 0.05, **p < 0.01, ***p < 0.001, ****p < 0.0001. Fig. S3

Fig. S4 Bone Turnover Markers Following PTH and Dietary Treatments in Male Mice. Serum markers of bone turnover including (A) procollagen I intact N-terminal or PINP and (B) C-terminal telopeptide or CTx from male mice fed a control (Con; 10 % kcal from fat) or moderately ‘high’ fat diet (hFD; 25 % kcal from fat) and treated with vehicle (Veh; closed circle) or parathyroid hormone (PTH; open square) for 5 days/wk. over 4 weeks. Each dot represents data from individual animal (n = 7–8). All results are expressed as mean ± standard deviation. Significant differences were established using 2-way analyses of variance (2-way ANOVA) with diet and treatment as independent variables, with multiple comparisons and post-hoc analysis using Fisher's LSD tests. Values of p < 0.05 were considered significant, with p-values depicted as *p < 0.05, **p < 0.01, ***p < 0.001, ****p < 0.0001. Fig. S4

Supplemental Table 1 The diets used for these experiments used purified diets including the AIN93M, custom ‘high’ fat diet (25 % kcal from fat, hFD) and respective sucrose matched control diet (Con) formulas. The Con and hFD are loosely based on the dietary standard set forth by the American Institution of Nutrition (AIN) to ensure adequate macro- and micronutrients, as well as vitamins and minerals (not shown). Supplemental Table 1

## CRediT authorship contribution statement

**Santosh Thapa:** Writing – review & editing, Writing – original draft, Project administration, Methodology, Formal analysis, Data curation. **Jake Newberry:** Writing – review & editing, Project administration, Formal analysis, Data curation. **S.V.V.S. Ravi Mangu:** Writing – review & editing, Project administration, Data curation. **Ron C.M. Helderman:** Writing – review & editing, Methodology, Formal analysis. **Ananya Nandy:** Writing – review & editing, Project administration, Methodology. **Cameron Fawcett:** Writing – review & editing, Methodology, Data curation. **Sasidhar Uppuganti:** Writing – review & editing, Methodology, Formal analysis. **Jeffry S. Nyman:** Writing – review & editing, Supervision, Methodology. **Elizabeth Rendina-Ruedy:** Writing – review & editing, Writing – original draft, Visualization, Validation, Supervision, Software, Resources, Project administration, Methodology, Investigation, Funding acquisition, Formal analysis, Data curation, Conceptualization.

## Declaration of competing interest

None of the authors have anything to declare.

## Data Availability

Data will be made available on request.
