# Supplementary figures and images for: Increased fatty acid availability improves the osteo-anabolic effects of intermittent parathyroid hormone (iPTH) in murine models
Source: Bone Rep. 2025 Nov 9;27:101887. doi: 10.1016/j.bonr.2025.101887 (PMC12661984; doi:10.1016/j.bonr.2025.101887)

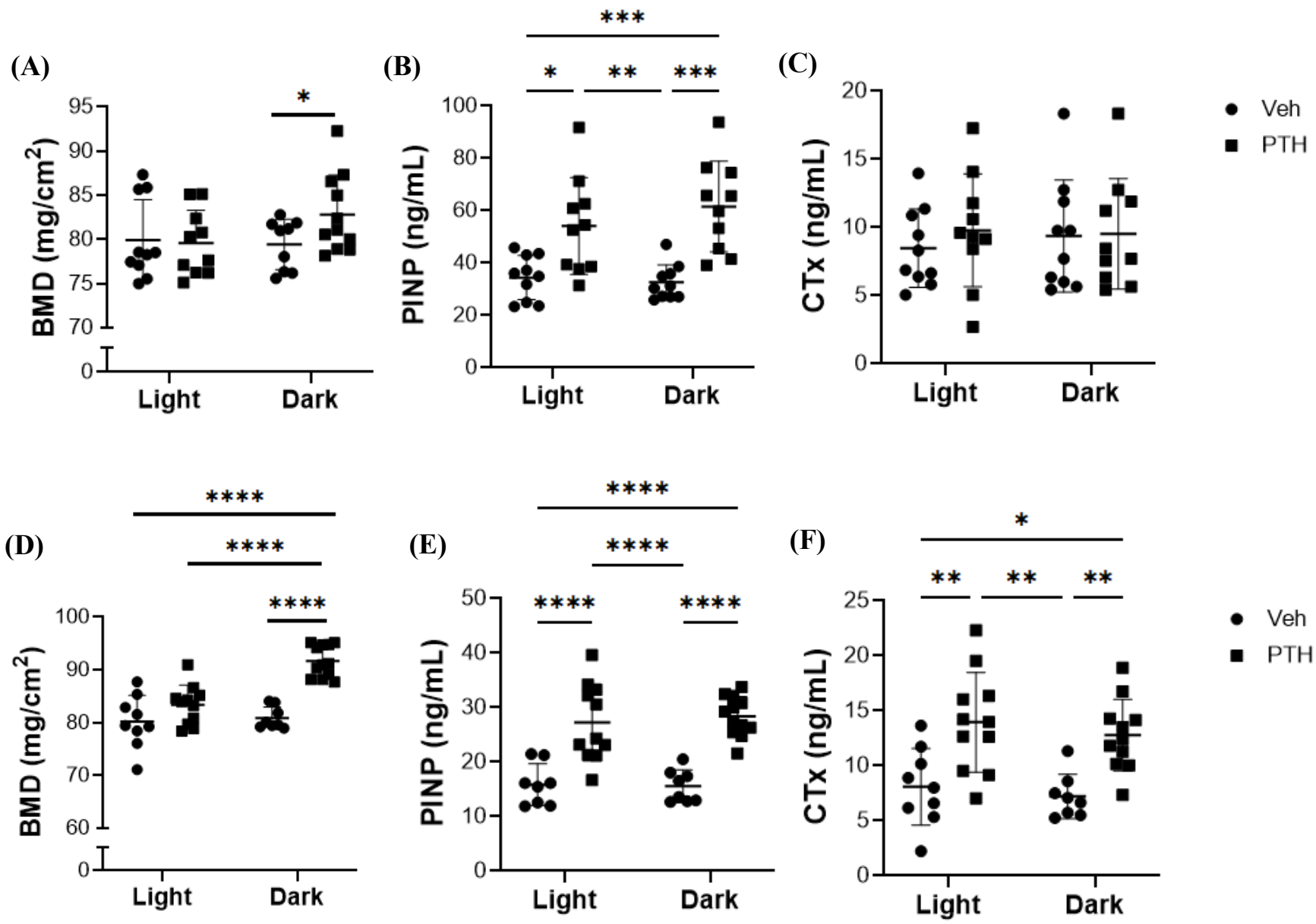

Supplement: Fig. S1 — Determination of Time-Related Efficacy during PTH treatment. Twelve-week-old C57BL/6N fe/male mice were fed on an AIN-93M purified diet and subcutaneously treated with vehicle (Veh; closed dark circle) or parathyroid hormone (PTH; closed dark square); 5 days/ wk for a total of 4 weeks. Mice received injections during their light cycle (Light) or dark cycle (Dark) under a red light. Dual-energy X-ray absorptiometry (DXA) results of bone mineral density (A) and (D) for female and male mice, respectively. Serum was isolated and used for bone turnover markers including procollagen I intact N-terminal or PINP (B and E) and C-terminal telopeptide or CTx (C and F) for female and male mice, respectively. Each dot represents data from individual animal where n = 7–11. All results are expressed as mean ± standard deviation. Significant differences were established using 2-way analyses of variance (2-way ANOVA) with diet and treatment as independent variables, with multiple comparisons and post-hoc analysis using Fisher's LSD tests. Values of p < 0.05 were considered significant, with p-values depicted as *p < 0.05, **p < 0.01, ***p < 0.001, ****p < 0.0001. [file mmc1.pdf]

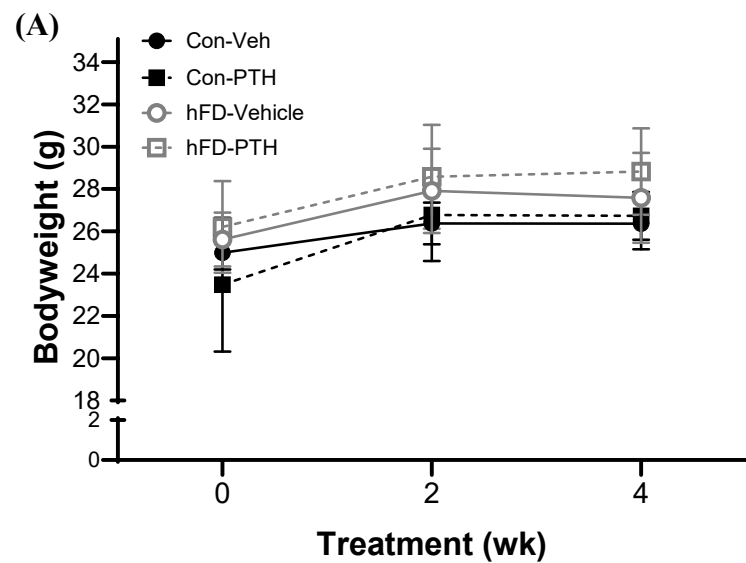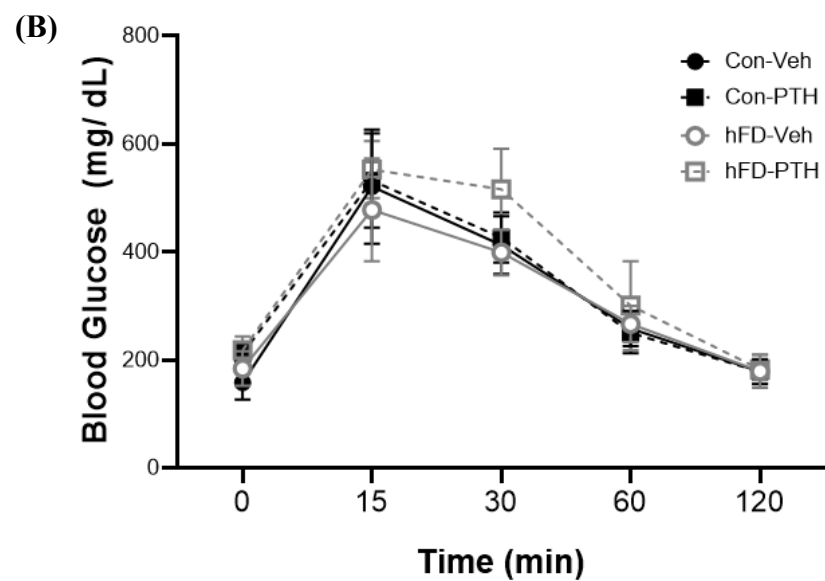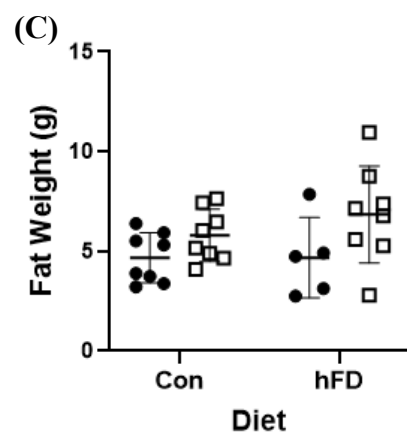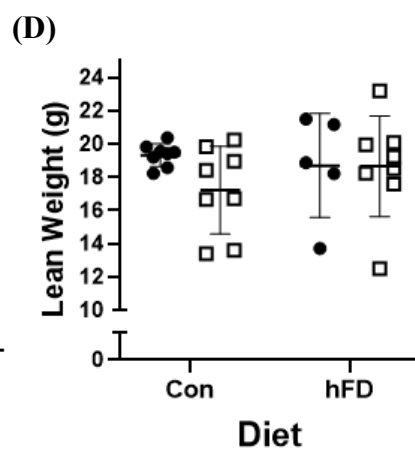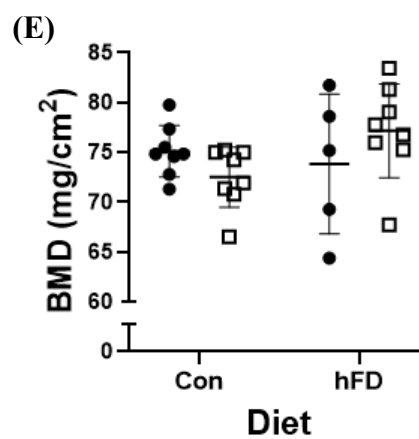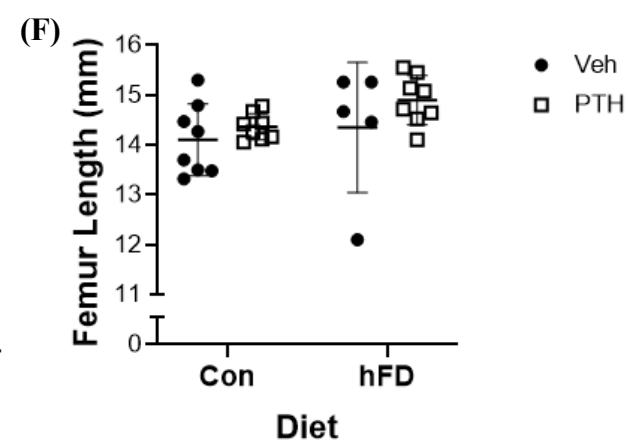

Supplement: Fig. S2 — Systemic Metabolism and Bone Characterization Following Dietary Treatment in Male Mice. Twelve-week-old male C57BL/6N mice were fed on a control diet (Con; 10 % kcal from fat) or moderately ‘high’ fat diet (hFD; 25 % kcal from fat) and treated with vehicle (Veh) or parathyroid hormone (PTH; 5 days/ wk) for a total of 4 weeks. Parameters include (A) bi-weekly bodyweight and (B) fasting, oral glucose tolerance test (GTT) a week prior to the study termination. Dual-energy X-ray absorptiometry (DXA) results of (C) fat weight, (D) lean weight, and (E) whole body bone mineral density (BMD). (F) Femur length was determined using digital calipers. All results are expressed as mean ± standard deviation. C–F, each dot represents data from individual animal (n = 6–10) treated with Veh (closed circle) and PTH (open square). Significant differences were established using 2-way analyses of variance (2-way ANOVA) with diet and treatment as independent variables, with multiple comparisons and post-hoc analysis using Fisher's LSD tests. Values of p < 0.05 were considered significant, with p-values depicted as *p < 0.05, **p < 0.01, ***p < 0.001, ****p < 0.0001. [file mmc2.pdf]

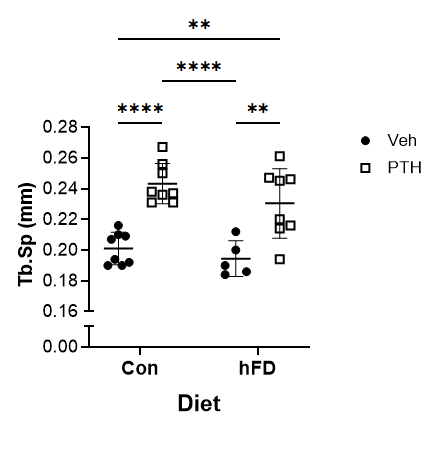

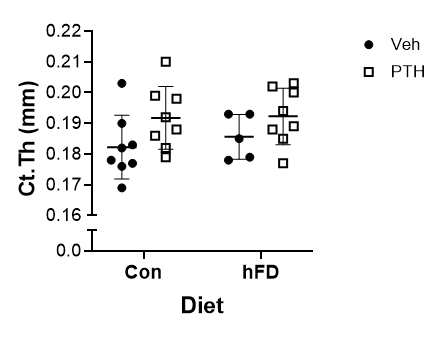

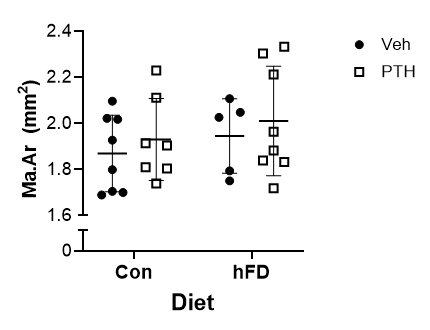

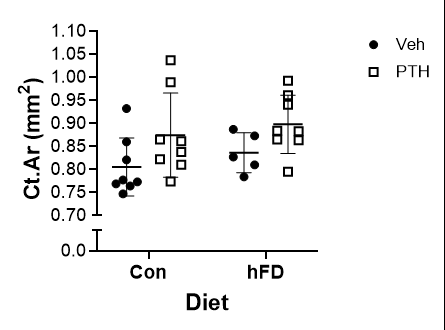

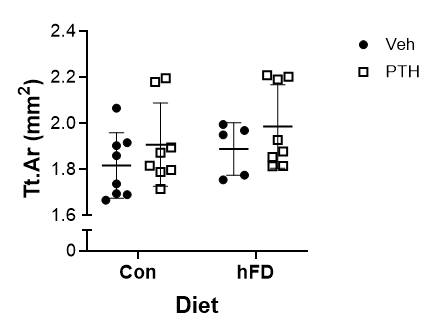

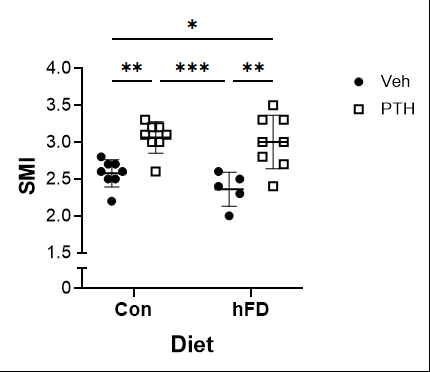

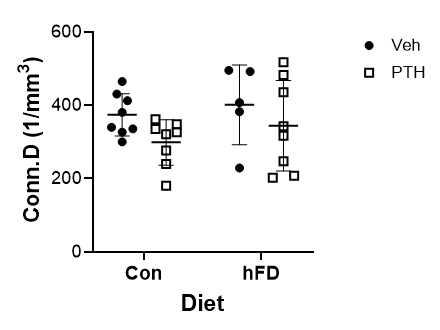

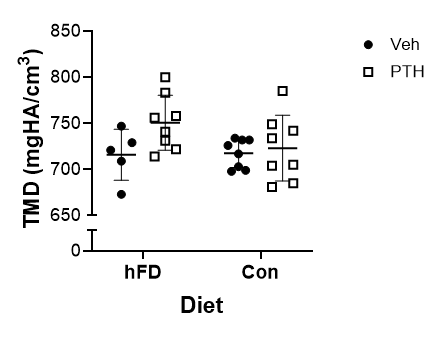

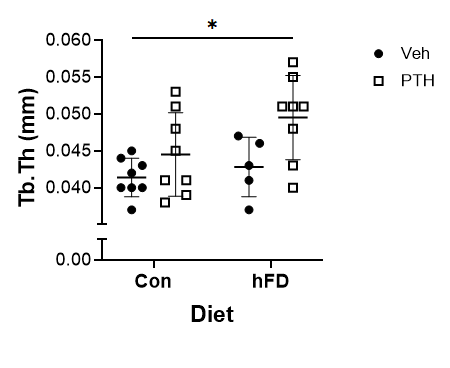

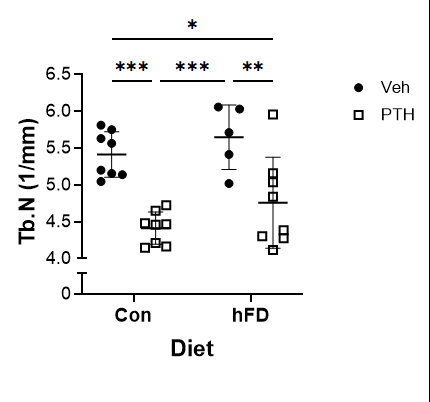

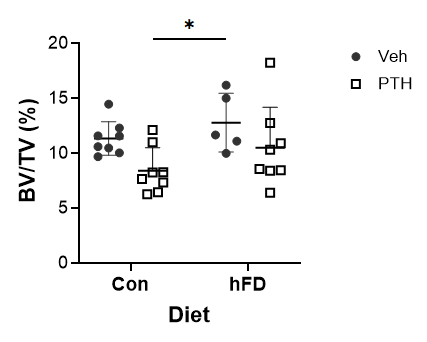

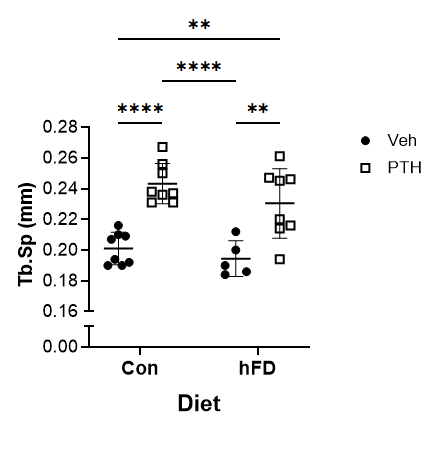

Supplement: Fig. S3 — Bone Microarchitecture in Male Mice Following PTH treatment during Dietary Modifications. Micro-computed tomography (μCT) analysis from the femur metaphysis of trabecular and cortical bone analysis of the femur mid-diaphysis in male C57BL/6 N mice fed a control (Con; 10 % kcal from fat) or moderately ‘high’ fat diet (hFD; 25 % kcal from fat) and treated with vehicle (Veh; closed circle) or parathyroid hormone (PTH; open square) for 5 days/wk. throughout 4 weeks. Trabecular parameters (A–G) include trabecular bone volume fraction, trabecular number (Tb.N), trabecular thickness (Tb.Th), trabecular separation (Tb.Sp), tissue mineral density (TMD), connectivity density (Conn.D), and structural model index (SMI). Cortical bone properties (H–K) include total cross-sectional area (Tt.Ar), cortical area (Ct.Ar), marrow area (Ma.Ar), and cortical thickness (Ct.Th). Each dot represents data from individual animal where n = 7–10. All results are expressed as mean ± standard deviation. Significant differences were established using 2-way analyses of variance (2-way ANOVA) with diet and treatment as independent variables, with multiple comparisons and post-hoc analysis using Fisher's LSD tests. Values of p < 0.05 were considered significant, with p-values depicted as *p < 0.05, **p < 0.01, ***p < 0.001, ****p < 0.0001. [file mmc3.docx]

(A)

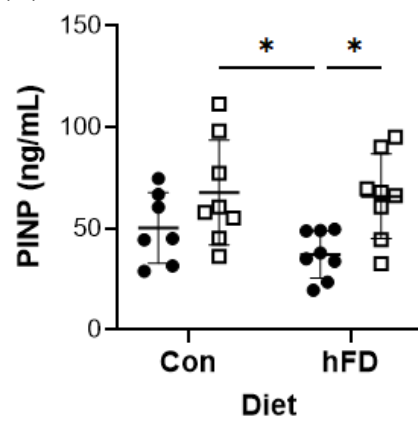

(B)

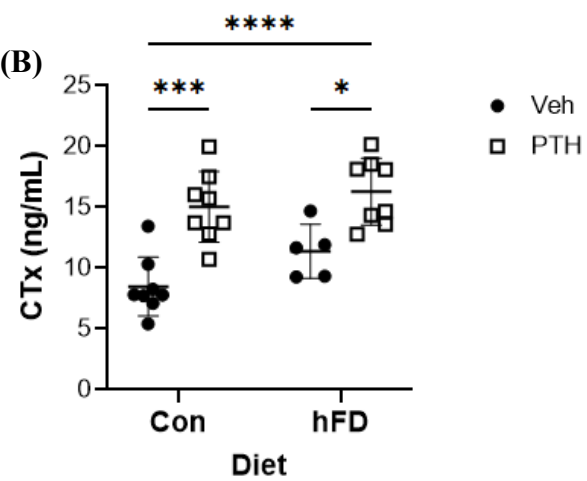

Supplement: Fig. S4 — Bone Turnover Markers Following PTH and Dietary Treatments in Male Mice. Serum markers of bone turnover including (A) procollagen I intact N-terminal or PINP and (B) C-terminal telopeptide or CTx from male mice fed a control (Con; 10 % kcal from fat) or moderately ‘high’ fat diet (hFD; 25 % kcal from fat) and treated with vehicle (Veh; closed circle) or parathyroid hormone (PTH; open square) for 5 days/wk. over 4 weeks. Each dot represents data from individual animal (n = 7–8). All results are expressed as mean ± standard deviation. Significant differences were established using 2-way analyses of variance (2-way ANOVA) with diet and treatment as independent variables, with multiple comparisons and post-hoc analysis using Fisher's LSD tests. Values of p < 0.05 were considered significant, with p-values depicted as *p < 0.05, **p < 0.01, ***p < 0.001, ****p < 0.0001. [file mmc4.pdf]
