# Supplementary material for: Increased fatty acid availability improves the osteo-anabolic effects of intermittent parathyroid hormone (iPTH) in murine models
Source: Bone Rep. 2025 Nov 9;27:101887. doi: 10.1016/j.bonr.2025.101887 (PMC12661984; doi:10.1016/j.bonr.2025.101887)
Supplement: Supplemental Table 1 — The diets used for these experiments used purified diets including the AIN93M, custom ‘high’ fat diet (25 % kcal from fat, hFD) and respective sucrose matched control diet (Con) formulas. The Con and hFD are loosely based on the dietary standard set forth by the American Institution of Nutrition (AIN) to ensure adequate macro- and micronutrients, as well as vitamins and minerals (not shown). [file mmc5.pdf]

		AIN-93M	high Fat (hFD)	Control
Protein	kcal%	14.7	20	20
	g/kg	*141.8*	*203.8*	*200.5*
Carbohydrate	kcal%	73.1	55	70
	g/kg	*720.7*	*553.1*	*706.9*
Fat	kcal%	4	25	10
	g/kg	*40*	*116.4*	*42.2*
kcal/g		3.85	4.18	3.85
Casein	kcal	560	800	800
	g/kg	*140*	*206.1*	*189.6*
Sucrose	kcal	400	275	275
	g/kg	*100*	*68.8*	*65.2*
Cornstarch	kcal	1983	1415	2024.8
	g/kg	*495.7*	*353.8*	*479.8*
Maltodextrin	kcal	500	500	500
	g/kg	*125*	*128.8*	*118.5*
Lard	kcal	/	789	180
	g/kg	/	*90.4*	*18.9*
Soybean Oil	kcal	360	225	225
	g/kg	*40*	*25.8*	*23.7*

Supplemental Table 1. The diets used for these experiments used purified diets including the AIN93M, custom 'high' fat diet (25% kcal from fat, hFD) and respective sucrose matched control diet (Con) formulas. The Con and hFD are loosely based on the dietary standard set forth by the American Institution of Nutrition (AIN) to ensure adequate macro- and micronutrients, as well as vitamins and minerals (*not shown*).
